# A novel applicability domain technique for mapping predictive reliability across the chemical space of a QSAR: reliability-density neighbourhood

**DOI:** 10.1186/s13321-016-0182-y

**Published:** 2016-12-03

**Authors:** Natália Aniceto, Alex A. Freitas, Andreas Bender, Taravat Ghafourian

**Affiliations:** 1 Medway School of Pharmacy, Universities of Kent and Greenwich, Anson Building, Central Avenue, Chatham, Kent ME4 4TB UK; 2 School of Computing, University of Kent, Canterbury, Kent CT2 7NF UK; 3 Centre for Molecular Science Informatics, Department of Chemistry, University of Cambridge, Lensfield Road, Cambridge, CB2 1EW UK; 4 School of Life Sciences, JMS Building, University of Sussex, Brighton, BN1 9QG UK

**Keywords:** QSAR, Applicability domain, P-gp, Prediction reliability, k-Nearest neighbour, dk-NN, Kernel density estimation, P-glycoprotein

## Abstract

The ability to define the regions of chemical space where a predictive model can be safely used is a necessary condition to assure the reliability of new predictions. This implies that reliability must be determined across chemical space in the attempt to localize “safe” and “unsafe” regions for prediction. As a result we devised an applicability domain technique that addresses the data locally instead of handling it as a whole—the reliability-density neighbourhood (RDN). The main novelty aspect of this method is that it characterizes each single training instance according to the density of its neighbourhood in the training set, as well as its individual bias and precision. By scanning through the chemical space (by iteratively increasing the applicability domain area), it was observed that new test compounds are successively included into the applicability domain region in such a manner that strongly correlates to their predictive performance. This allows the mapping of local reliability across different locations in the training set space, and thus allows identifying regions where the model has low reliability. This method also showed matching profiles between two external sets, which is an indication that it performs robustly with new data. Another novel aspect in this technique is that it is paired with a specific feature selection algorithm. As a result, the impact of the feature set used was studied from which the top 20 features selected by ReliefF yielded the best results, as opposed to using the model’s features or the entire feature set as commonly done. As the third novel aspect, in this work we propose a new scoring function to help evaluate the quality of an applicability domain profile (i.e., the curve of accuracy vs the applicability domain measure in question). Overall, the RDN showed to be a promising method that can correctly sort new instances according to predictive performance. As a result, this technique can be received by an end-user as proof of concept for the performance of a QSAR model in new data, thus promoting the user’s trust on the QSAR output.

**Electronic supplementary material:**

The online version of this article (doi:10.1186/s13321-016-0182-y) contains supplementary material, which is available to authorized users.

**Graphical abstract:**
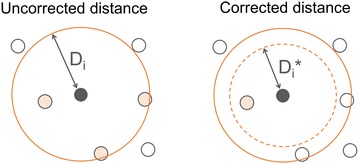
.

## Background

Any chemistry-response relationship model needs to demonstrate not only good accuracy but also reliability of external predictions. To address the latter, it is necessary to establish chemical space boundaries where the model has reliable and defined performance. These boundaries are commonly known as the applicability domain (AD), and define the extent to which a quantitative structure–activity relationship (QSAR) model (reliably) tolerates new compounds [1, 2]. As pointed out by Eriksson [1], end users of the model will only trust the model’s predictions if they have evidence that the chemical space used for training matches the one of newly tested compounds.

There are several reviews and comparative studies on AD methods available in the literature [3–8], which focus on either distinguishing inliers from outliers, or high accuracy compounds from low accuracy compounds. Contrarily to the modelling task where a response variable can be used to assess the predictive ability of the model, there is no response variable for the *true* inclusion in the AD given its subjective nature. As a consequence, the characterization of a model’s AD is exploratory by nature. So, a main question must be answered whenever any characterization of this sort is put in place: *Will this applicability domain be useful in identifying reliable predictions in new queries?*

So far, there is no clear focus in the community for assessing whether an AD established with training data is able to successfully point out if a new prediction may be accepted or not. QSAR modellers often implement any given AD method and merely determine the portion of external data (and its accuracy) falling within the established boundaries, without any assessment of the ability of the AD boundary to differentiate between “acceptable” and “unacceptable” new predictions. Therefore, it is impossible for the user to validate and trust an arbitrary threshold. Applying a threshold and showing that inside that threshold, data have higher accuracy as carried out in some previous work [8, 9] provides useful information, but ignores the possibility of localized inner “holes” in the chemical space where the model is unreliable.

As mentioned before, when defining the AD there is no way of objectively determining the accuracy of forecasts on inclusion/exclusion criteria of new queries within the AD. However, one is able to estimate the utility of a certain AD in a real world scenario by applying it to naïve data.

A useful AD should relate similarly to the predictive reliability in the training set and in an external dataset. To illustrate this notion, let us consider an AD that shows a constant degradation of accuracy with increasing distance to the AD core (here the term “core” can be interpreted as the sum of one or more centroids in the AD, where predictive confidence is maximum). Even though this apparently depicts data reliably across the structure landscape, when applied to an external dataset, the relationship between accuracy and distance-to-model values output by the AD technique gets inverted, which renders this AD useless given its unpredictability when handling new data. This scenario is demonstrated in the “Results” section, using a kernel density estimation (KDE) AD method. Ideally, a valid AD would be sufficiently robust and not affected by changes in dataset, thus allowing the maintenance of the general AD premise by which a model’s performance degrades as the queried instances get farther away from the training chemical space.

The majority of currently available AD methods usually focus on a single property of the data, for example similarity, descriptor range, density or response-range (or ensemble-range). A list of methods across categories can be found in the literature [10]. However several works support the need to combine different properties (such as response, density and similarity) to achieve a reliable characterization of a model’s AD [10–12]. Furthermore, most methods address data globally (e.g., location with respect to global feature span or density across global feature set), even though it is well established that the modelled data can exhibit very different properties in a local level versus the global level. This has been explored recently by Sahigara et al. [12] in an attempt to distinguish predictions according to their reliability. This work shows a novel approach where local AD is tailored according to the data density at specific locations across the model space. This allows a detailed characterization of the local nature of the modelled data. However, in this approach, locations in the chemical space are characterized only according to local data density, whereas we hypothesize that a model’s AD is a function of, not only the local data density, but also of the local reliability, i.e., the net effect of local precision and bias.

**Fig. 1:**
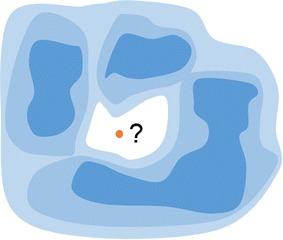
Applicability domain across two projected variables. *Darker regions* correspond to highly dense and reliable regions

In this work we propose a new AD method which combines two other previously published methods—the STD method [13] and the k-nearest neighbours density (dk-NN) approach [12]. We have named this technique reliability-density neighbourhood (RDN). This AD technique maps external predictions with regard to distance to the model space while taking into account the reliability of nearby training instances, thus accounting for the variable nature of different data localities both in terms of multi-dimensional localization (as multiple dimensions are input into the distance calculation) and predictive reliability. Here, we suggest a reliability measure that is the net result of two distinct effects, bias and precision. Figure 1 shows a schematic depiction of the RDN AD, where density and reliability are mapped across chemical space showing densely populated and more reliable areas in darker blue, transitioning into white regions of sparse and/or unreliable data. The other novel aspect introduced with this method is the optimization of the set of molecular descriptors used as input to compute neighbour distances. This is another important feature to take into consideration since the AD is only as explanatory as the ability of its molecular features to chemically distinguish mispredictions from correctly predicted instances. It is important to highlight that the prediction task is independent of the AD implementation and outcome.

Hence, in this work we introduce three novel aspects to the topic of AD characterization: (1) exploiting the role of feature selection in building a high-quality AD, (2) introducing a new AD technique which takes into account the individual characteristics of each location across the training space, namely data density, bias and precision, and (3) introducing a new scoring scheme to evaluate the robustness and qualitative value of AD techniques. As a result, we propose the importance of evaluating AD robustness for the first time. An R package with the implementation of RDN is available at https://github.com/machLearnNA/RDN, allowing an easy and straightforward installation and use, directly from the R environment.

## The algorithm

To better support the utility of this new technique we will describe the density k-NN (dk-NN) approach proposed by Sahigara et al. [12], which was the basis from which we developed the herein proposed method; we will subsequently build on this explanation to transition into the RDN algorithm. The novel parameters and their contribution to the overall mechanism of this new technique will be discussed.

The dk-NN AD proposed by Sahigara et al. [12], uses the k-NN principle associated with the concept of adaptive kernel techniques in KDE to detect local neighbourhoods within the data. This approach capitalizes on the notion that any given dataset can have a very different behaviour at the local level when compared to the global behaviour. In this method, the average Euclidean distance (using standardized descriptors) between each training compound and its k nearest neighbours is computed, which is used to calculate a reference value (RefVal) set at Q3 + 1.5 × IQR (also known as the Tukey’s outlier fence [14]), where Q3 is the 3rd quartile and IQR is the interquartile range calculated as the difference between the 3rd and the 1st quartiles of the list of average distances. The neighbourhood width threshold for *each* individual training compound (*D*_*i*_) is then calculated as the average distance to all its training neighbours with distance values closer or equal to the RefVal. By establishing different local thresholds, this addresses the variation of data density across the dataset.

As we realised the dk-NN AD is limited only by the degree of emptiness of the different regions occupied by the data (i.e., a sparse region will render its occupiers a smaller distance threshold, under a given established k value, as these instances will have no neighbours within the average overall distance to the k-th nearest neighbour), it would be logical to tailor each different neighbourhood (i.e., coverage width around each training instance) according to their reliability. To measure reliability we used both bias and precision as explained below.

**Fig. 2:**
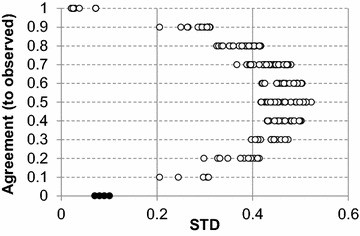
Relationship between agreement and ensemble standard deviation in the P-gp IV dataset. In this case STD translates into accordance among a set of predictions (i.e., precision), whereas Agreement refers to the level of bias in that set of predictions

Following the theoretical principle that an ensemble (set) of models, *M*, will have a high degree of accordance and consequently a smaller standard deviation (STD) for more reliable predictions, one would expect that regions where a clear, smooth structure–activity relationship is found would generate more robust predictions that are less susceptible to changes in the learning task (i.e., changing the data partition within the ensemble). Alternatively, regions with a less stable landscape will rely greatly on the data partition used, thus generating larger differences between different models [15]. However, as STD values only measure the level of precision, the rate of agreement between the set of predictions and the real responses needs to be used to overcome cases of systematic bias towards an incorrect classification. More precisely, a systematic bias occurs when the majority of predictions are close to each other, but all are wrong, as represented by the black instances in Fig. 2. These predictions would be captured by the algorithm as high reliability predictions if only an STD correction was used. As a consequence, the combination of bias and precision is an appropriate correction factor for reliability, *W*_*i*_.

Taking this notion into account, we have built upon the dk-NN algorithm to create the RDN AD method herein proposed by introducing a weighting term defined in Eq. 1, which measures the reliability associated to each training instance.

1 $$W_{i} = \left( {\mathop {1 - \sqrt {\frac{{\sum\nolimits_{m = 1}^{M} {\left( {\widehat{{y_{i,m} }} - \overline{{y_{i} }} } \right)}^{2} }}{M - 1}} }\limits_{\text{STD}} } \right) \times \mathop {\frac{{\left| {Y_{i} \cap \widehat{{Y_{i} }}} \right|}}{M}}\limits_{\text{agreement}}$$

where the first term (1-STD) measures precision and the second term (agreement) measures bias. In this equation, the weighting factor $\widehat{{y_{i,m} }}$ is the predicted response for compound *i*, output by model *m,* among *M* models in the ensemble; $\overline{{y_{i} }}$ is the average prediction output by the ensemble model; $Y_{i}$ is the experimental response; and $\widehat{{Y_{i} }}$ is the prediction output by the QSAR model. As STD and agreement take values from 0 to 1, *W*_*i*_ will also take this range of values.

**Fig. 3:**
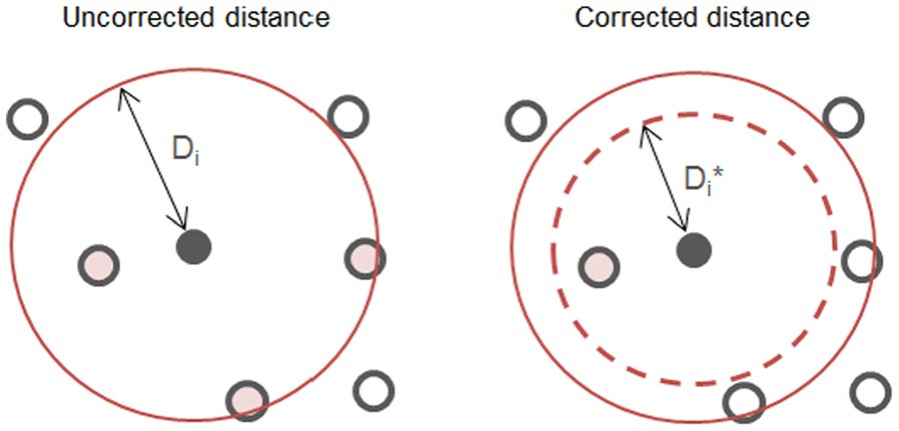
Scheme of the reliability correction of the distance *D*_*i*_ attributed to training compound *i*. The sphere’s radius, *Di*, will be decreased proportionally to the reliability of compound *i*. For example, if (1 − STD) × agreement is 80%, *D*_*i*_ will be reduced by 20% of its initial value, which means that the 2 of the initial 3 external instances that were covered by compound *i* will end up outside the neighbourhood coverage area supplied by this training compound

**Scheme 1:**
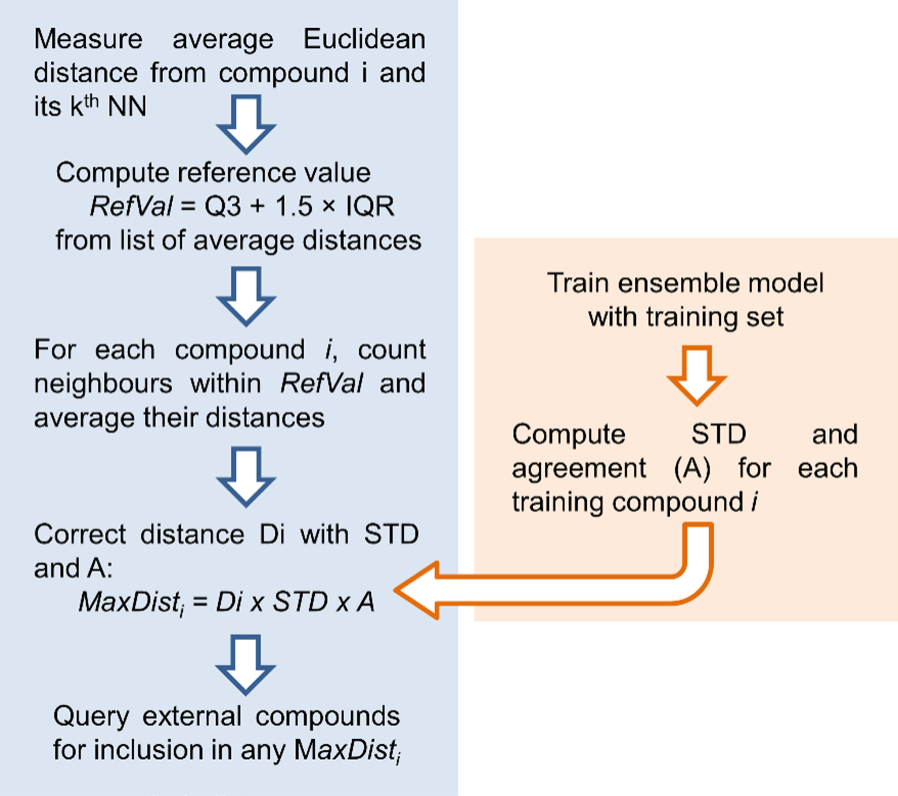
Pseudo-algorithm of reliability-density neighbourhood (RDN) applicability domain technique

For each training instance *i*, *W*_*i*_ will be multiplied to the respective threshold distance *D*_*i*_, calculated as previously explained. As STD is the deviation among an ensemble of predictions, 1 − STD is the precision rate. A high 1 − STD value, which translates into a high precision, will contribute to a large *W*_*i*_, and consequently to a small reduction of *D*_*i*_. As for the agreement term, increasing values translate into a decreasing level of bias. As such, a large agreement will entail a small penalization to *D*_*i*_. To illustrate the use of *W*_*i*_, the space (neighbourhood) covered by a given training point will be penalized proportionally to its degree of unreliability, i.e., for STD = 70% and agreement = 35%, a reliability of 10.5% is obtained, which leads to a 89.5% reduction of coverage attributed to its training instance. In a contrasting scenario, for a high reliability of 98% (STD = 1%; agreement = 99%), this will lead to a 2% reduction of the neighbourhood span (threshold). The effect of correcting neighbourhood distances for their reliability is demonstrated in Fig. 3. The complete flow of the described RDN algorithm is summarized in Scheme 1.

The success of addressing local bias and precision, as well as local distance to training has been demonstrated by Sheridan [11]; however they have sorted the data into several bins, which renders comparative analysis and the implementation of the AD rather difficult. A continuous performance characterization should allow the localization of gaps in the data/model’s chemical space in a more user-friendly way.

As the obtained individual thresholds associated with each training instance depend on the Euclidean distance between compounds, which in turn depends on the descriptors used, we propose pairing this AD technique with a prior feature selection routine. We have chosen ReliefF, originally proposed by Kononenko et al. [16], as this algorithm searches for a feature set that maximizes the separation of classes in the response variable within local neighbourhoods [17]. ReliefF has been shown to detect relevant features even in very crowded (feature-wise) datasets, whilst being resilient to noise [18, 19]. The appropriateness of this algorithm for this end can be justified by the fact that this feature selection method has 3 paramount properties with respect to AD definition: (a) it evaluates descriptors solely on their individual ability to separate classes; (b) it takes into account the local neighbourhoods when evaluating each feature; (c) identifies useless/irrelevant features that would only contribute with noise [20]. Regarding the first properties, while ReliefF allows the selection of highly correlated features, its performance is unaffected by the existence of correlation itself [21] which, contrarily to QSAR modelling, is expectedly a desirable feature for a successful AD as highly correlated features turn out to be complementary in chemical space coverage.

Considering that a QSAR model is focused on distinguishing between two different responses, and its AD is focused on discriminating between correct and incorrect predictions, it is expected that the molecular descriptors that are best suited for the former will not necessarily be the most appropriate for the latter, as previously suggested [11]. In fact, Sheridan et al. [22] have shown that descriptors used to define the model’s boundary do not have to coincide with the descriptors used to build that same model. Furthermore, note that an AD technique which does not rely on the features used by the QSAR model allows comparable implementation in both the so-called transparent methods (e.g., decision trees) and “black box” methods (e.g., artificial neural networks). As a result, the herein proposed AD method is paired with the ReliefF routine for feature selection.

## Methods

### Building of the QSAR model

In order to evaluate the performance of the currently proposed AD a dataset of P-glycoprotein (P-gp) substrates and non-substrates, compiled from data in the Metrabase database (accessed on October 2014, www.metrabase.ch.cam.ac.uk/), was used. Every compound with at least one reference supporting it as a substrate was considered as such.

A decision tree was trained using 60% of data (training [TR] set), optimized using 20% of the data (internal validation [IV] set), and tested on the remaining 20% (test [TE] set) by random allocation of compounds into these sets. Training was done using J48 in Weka 3.6, and optimization was done with respect to the feature selection method which was considered optimal according to highest IV performance. Five feature selection routines were applied to 334 descriptors calculated from ACD/labs logD suite v12.5 and MOE 2013. Briefly, two types of feature selection approaches were used: filter and wrapper methods. Filter methods rank each feature according to a given objective function (e.g., correlation to response variable, inter-feature correlation, etc.), while wrapper methods evaluate and select features which lead to the best predictive performance by associating a filter method with a machine learning algorithm (represented by a hyphen connecting both algorithms) [18]. The filter methods used were greedy search (GS), genetic algorithm (GA) search and ReliefF; and the wrapper methods used were J48-GA and random forest (RF)-GA. (for experimental details refer to the literature [23]). From those, the J48-GA wrapper method was selected for model (decision tree) building as it generated the feature set associated with the highest IV performance. The trained decision tree was used to produce class predictions in the form of probabilities, which were later used to evaluate AD performance. Note that the feature selection task undertaken within the model building process (described under this subsection) must not be mistaken for the feature selection role in establishing AD characterization. These two are separate and independent tasks.

### Feature selection in AD characterization

To establish an optimal feature set utilized in the RDN algorithm, more specifically in the calculation of the Euclidean distance between the compounds in the P-gp dataset, different thresholds of feature ranking using ReliefF were applied, namely the top 20, 50, 100 and 200 features as well as the entire feature set of 334 molecular descriptors. This led to five feature sets that were tested in the original dk-NN algorithm. For comparison, the J48-GA features used to train the QSAR model were also used, as it is a common practice to use the model’s features to describe the AD. RDN was not used to assess the effect of the descriptor sets as this would introduce additional noise to the system (due to different variables in play) and could confound the comparison between feature sets. As dk-NN takes solely into account the Euclidean distances between compounds, this allows a more straightforward observation of the effect of the feature set. Furthermore, a selection of the best feature set candidate(s) in RDN would increase the risk for parameter overfitting.

To diminish the impact of local solutions that are known to happen, for example with GA [24, 25], five feature selection routines were initiated from different points of the dataset and both ReliefF feature ranks and J48-GA feature frequencies were averaged so that each feature had an average rank/frequency value. Both methods were carried using Weka 3.6. ReliefF settings were numNeighbours = 10 (following empirical default [17, 20]) and sigma = 2 [17]. For J48-GA feature selection GeneticSearch was the search method with parameters: crossoverProb = 0.8, maxGeneration = 100, mutationProb = 0.01, and Population size = 100, as usually implemented [24, 26].

From this stage the two best candidates were selected for further testing with RDN.

### Consensus standard deviation (STD) applicability domain

Even though the STD measure was embedded in the RDN algorithm as part of the correction factor, this is a standalone AD method that has obtained excellent performance in sorting predictions according to their reliability. As a result, we used STD as our gold standard method against which RDN was compared [6, 7, 13, 27]. Note however that we will also report the results of dk-NN and KDE methods for comparison reason (methods explained further below).

For the implementation of the STD method, a tenfold bootstrap routine was performed in which, at each fold, 80% of the training data was randomly sampled (with replacement) to train a J48 model. This resulted in 10 decision trees which were used solely to produce reliability estimates in the form of overall deviation among the ten sets of prediction, while class predictions were performed separately by a single tenfold cross validated model. The STD value was calculated for each compound according to Eq. 2 [28]

2 $$STD = \sqrt {\frac{{\sum {(y_{m} - \bar{y})^{2} } }}{N - 1}}$$

where $y_{m}$ is the class prediction from model *m* and $\bar{y}$ is the average of all predictions output by *N* models, relatively to any given compound.

Contrarily to the QSAR model whose output is ultimately qualitative (an instance is assigned to the class of highest probability), we use the actual value of the probability towards the quantification of reliability. Consequently node calibration by Laplace smoothing (for a detailed outlining see [29]) has been used during the training of the ensemble model. Laplace estimate compensates for the node size, thus preventing overly optimistic probabilities at very small nodes.

### Reliability-density neighbourhood applicability domain

**Fig. 4:**
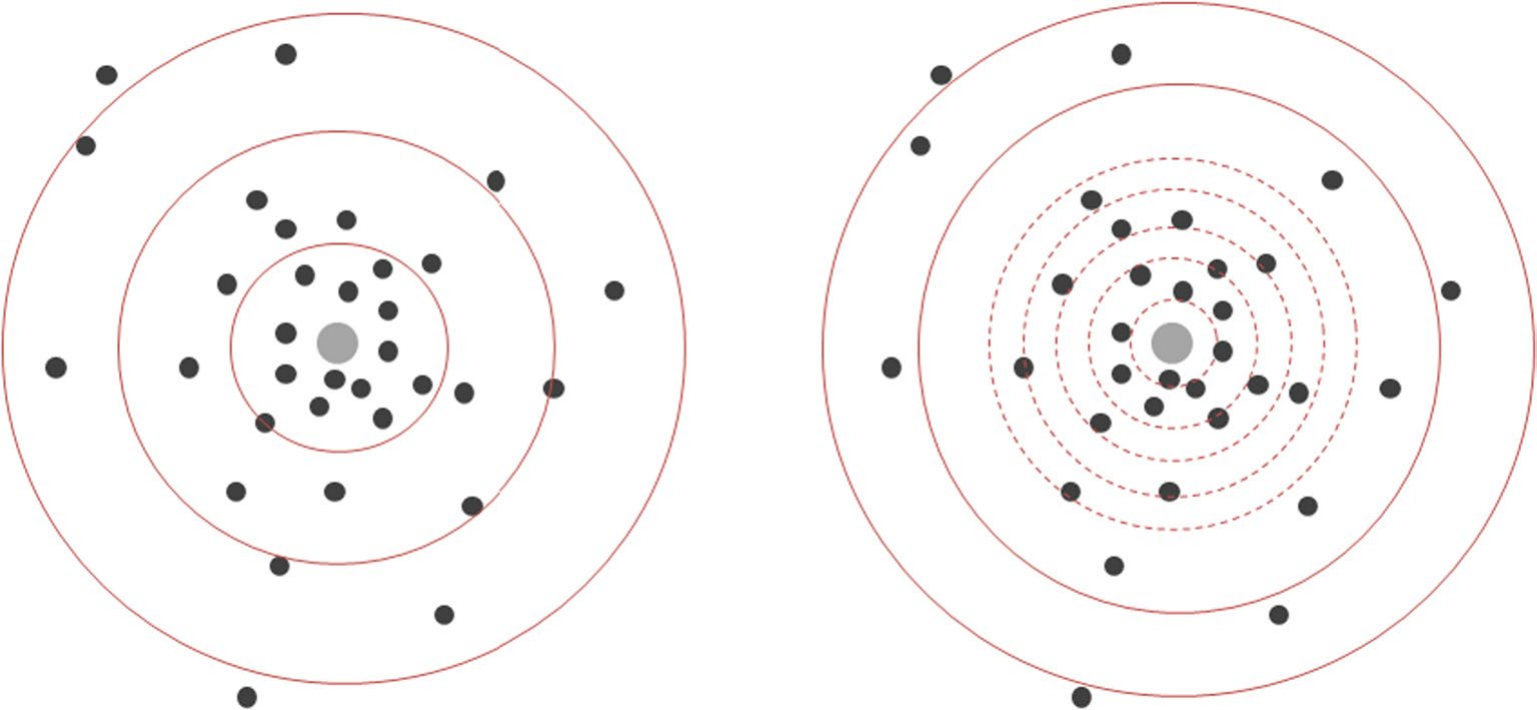
Schematic representation of the difference between the RDN algorithm without (*left*) and with (*right*) distance step adaptation. The *grey point* represents a training instance, and the *black points* depict external instances scattered across a 2D projection of the 20 molecular feature matrix. Smaller increases in radius around the training instance in* grey* increase sensitivity in measured accuracy across the AD landscape

The RDN AD was implemented as described in “The algorithm” section, being run iteratively at increasing k values, ranging from 1 to 65 nearest neighbours (NN), which corresponds to approximately 100% coverage of the data (as obtained empirically). This allows to scan the chemical space from denser areas to sparser areas. Our preliminary results showed that using the distance step size to the first NN directly was not ideal as the AD RefVal led to a too wide an AD (with more than 50% of data falling within the nearest 2–3 neighbours region). This is because this region is more densely populated thus being highly sensitive to even small increases in the distance threshold (see Fig. 4). Therefore, it is necessary to make sure that the initial neighbourhood thresholds increase slowly. Then, as the AD boundaries get larger, it is affordable to have larger distance increases at each step. To this end, the RDN algorithm was run at a third of the determined neighbourhood distance from k = 1–30, then half of the neighbourhood distance was used for k = 31–40, and finally for k values >40 the distance was used directly as computed. However, this is an arbitrary setting that can be tailored according to the user’s needs, and different distance step sizes can be used to obtain different levels of detail in the plots of accuracy *vs* percentage of data in the AD. As exemplified in Fig. 4, implementing an initial smaller step size in the increase of the distance thresholds (right-hand side) allows a slower inclusion of data into the AD, which consequently improves sensitivity at the inner core of the model.

As originally implemented in the dk-NN algorithm, a query must fall within the neighbourhood threshold of at least one training instance in order to be considered inside the AD. This prompted the assessment of the impact that the number of required training neighbours has on the overall performance of the AD. To do so, the algorithm was tested with different minimum required k values which offer coverage to new instances, ranging between 2 and 30.

For the calculation of the RDN AD profile, *W*_*i*_ (Eq. 1) is calculated for each training instance to correct their neighbourhood radius distance according to their level of precision and bias. For the P-gp model, STD was calculated from the deviation between a tenfold bagged decision tree ensemble, as shown in Eq. 2. Regarding the values of agreement, these were calculated by determining the frequency of predictions in the ensemble which were correct (i.e., matching the observed class).

## Comparison with dk-NN and KDE AD methods

For comparison, STD and dk-NN methods have been implemented as they both are integrated in the RDN algorithm. The implementation of both was done as described earlier. Additionally, kernel density estimation (KDE) has been used for its specific features which address data from a different perspective. Similarly to k-NN, KDE addresses data density, however the former focuses on local neighborhoods whereas the latter addresses overall data density across descriptor space. Since RDN accounts for both density and predictive reliability, it is worth evaluating both density in chemical space (both locally and globally) and response distribution separately. KDE was computed using KernelDensity within the sklearn python module, in which a Gaussian kernel was used and the bandwidth was selected from an online platform (http://176.32.89.45/~hideaki/res/kernel.html) of bandwidth optimization created by Shimazaki and Shinomoto [30]. The implementation of KDE followed the procedure outlined elsewhere [3]. The density distribution model was established from the first principal component obtained from the training set, and the IV and TE sets were matched against it to test the hypothesis of density being correlated with predictive accuracy (i.e., accuracy decreases with decreasing density).

Furthermore, as the P-gp model was built using a decision tree learner it is worth monitoring misprediction occurrence with respect to chemical span in the decision tree’s branches. This analysis aimed at identifying any trends within the decision tree chemical space subpartitions.

### Quantitative comparison between AD methods

In order to establish which AD method yields the best performance, we propose a scoring function that aims for a quantitative, objective comparison between methods. This scoring function evaluates two features: (1) robustness, by measuring the similarity between the AD profiles of two external datasets, and (2) proximity to a smooth descending AD profile (accuracy vs the AD-produced measure of prediction confidence).

This scoring function is meant for the scoring of *continuous* ADs, not being suited for *in*–*out* binary type approaches. As any AD method is only reliable if it is robust when submitted to different subsets of the same dataset, this AD scoring function will quantify the ability of an AD to produce the same outcome in two different external datasets Y and Z. In an ideal scenario, where the AD of a model is mapped in a robust manner across the training data, Y and Z would yield two perfectly matching curves of accuracy *vs* distance-to-model (DTM). This indicates that the model’s reliability readout (i.e., trend between predictive performance and the AD measure) is not being affected by the specific dataset being evaluated, but instead the AD is robust enough to describe the predictive reliability across the data. Additionally, in the curves for both datasets Y and Z, the accuracy inside the AD boundaries should decrease steadily as a function of DTM, as it is theoretically expected that a model’s performance will degrade as the distance to training space increases. Equation 3 quantifies both aspects and produces a final score.

3 $$AD score = \frac{1}{{F_{{added, \left[ {1;P} \right]}} }}\mathop \sum \limits_{i = 2}^{P} WP_{i} \times \left| {y_{i} - z_{i} } \right| + WP_{i}$$

In this AD scoring function, (*y*_*i*_ − *z*_*i*_) quantifies the accuracy difference at each AD distance, *i*, and WP_i_ stands for weighted slope mismatch penalty at distance *i*, which measures the mismatch between curves direction at each distance interval. This will cover the entire curve of measured ACC versus AD measure across all points, P. We have used a weighted measure for the slope mismatch explained below. More specifically, as each distance point is associated with a given amount of newly added instances (N_added_) into the AD, the slope mismatch penalty is weighted according to how many instances have been added at a given distance interval (Eq. 4).

4 $$WP_{i} = SMP_{{\left[ {i,i - 1} \right]}} \times \frac{{N_{added,i} \left\{ {y + z} \right\}}}{{N_{total} \left\{ {y + z} \right\}}}$$

**Fig. 5:**
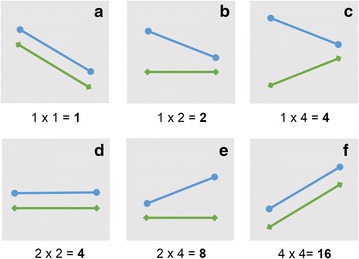
Representation of the different possible slope mismatch penalties, organized from the most desirable (ideal) scenario in **a** to the least desirable scenario in **f**

As the AD is expanded (DTM is being increased), the directions of the two curves are monitored using a term that penalizes slope mismatch between the curves, the slope mismatch penalty (SMP). We have set a qualitative penalty scheme that differentiates the various types of mismatch, described as follows (see Fig. 5).

The slope, m, of any segment in an AD curve (between distances *i* and *i* − *1*) can be *m* = 0, *m* > 0 or *m* < 0. Considering the requirement that accuracy should decrease with respect to distance-to-model, it is reasonable to consider m < 0 as the desirable case, m = 0 as less desirable and m > 0 as the least desirable case. As such, a multiplicative penalty of 1 (i.e., no penalty) has been attributed to a negative slope and it doubles consecutively for a null slope and a positive slope (i.e., 2 and 4, respectively). This set of penalties was optimized to allow a correct scoring of a positive control (a visibly highly similar pair of curves) and negative control (a visibly highly dissimilar pair of curves), i.e., a lower positive control score. To compare two corresponding pair-wise segments each segment on both curves is attributed a penalty according to its individual slope. The resulting product of the individual penalties of those two equivalent segments between *i* and *i* − *1* of the curve corresponds to SMP_*i*_. The various possible scenarios are exemplified in Fig. 5, where they are organized from the most desirable to the least desirable (from A to F, respectively).

Weighting of SMP by the amount of data points that are added to the applicability domain with each step of increased distance-to-model allows accounting for different local densities, which is necessary considering that a shift in the slope direction is more significant if it is caused by the addition of, for example, 50 new data points than by 2. As the scoring function is comparing each pair of corresponding points in both Y and Z curves, the total of instances under such pair of points are added together and divided by the total instances of both, to allow comparison between AD techniques that produce a different amount of distance-to-model points.

In addition, the absolute difference of accuracy (|*y*_*i*_ − *z*_*i*_|) under the same distance-to-model value (X-axis) is also included in the AD scoring function. This corresponds to the underlying concept of the Fréchet distance commonly used to measure curve similarity [31]. However, this is not a decisive aspect since a shift in absolute accuracy values will not have any impact in the decision of accepting or rejecting any given prediction, as long as the AD curves match in shape (i.e., the highest accuracy occurs at the same region for both curves). As a result, this is included with the sole purpose of allowing to differentiate between two pairs of curves where, in each pair, both curves have exactly the same shape within the pair, but one pair shows larger deviation of absolute accuracy values. To prevent this parameter from having a large impact on the total score (which would be inappropriate), it was added as coefficient of WP, as depicted in Eq. 3.

Lastly, as different AD techniques cover a different amount of data with their first iteration, which can be regarded as the AD’s core, it is desirable to differentiate between AD techniques according to their resolution at the model’s core. It is more useful to cover 5% of the total data with the first iteration than 50% of the data, as the user has no information regarding the accuracy versus distance relationship across that portion of the data. As a result, the final sum across all distances *i* is divided by the fraction of covered data from the first iteration to the last (F_added_); as this value approaches 1, the resolution at the model’s core increases, and the final sum is increasingly less inflated.

### Testing on benchmark datasets

To exclude the possibility of an exceptional performance under the P-gp dataset, two benchmark classification datasets were tested: the Ames mutagenicity dataset (“Ames levenberg” model entry, referred to as “Ames” from now on) and the CYP450 inhibition dataset (“CYP450 modulation e-state” model entry, referred to as “CYP450” from now on). To avoid any additional bias, the datasets were previously modelled [28] and the predictions were used as provided at the OChem QSAR modelling repository (https://ochem.eu/home/show.do). To allow testing the robustness of the AD profile, the validation datasets retrieved from OChem were split into two. Therefore, in this work, AD was evaluated in the P-gp model using the IV and TE, and the AD of the two models of benchmark datasets was assessed by splitting the provided external dataset into two sets of data. The Ames dataset comprised a training set of 4358 compounds, and two external sets of 1089 and 1090 compounds. The CYP450 dataset comprised 3743 training compounds, and 1870 compounds in each of the external test sets.

To maximize direct comparability, the source of the feature set used in every AD technique implemented for each dataset was kept fixed. As the purpose of this study is to validate the observed profile with the P-gp model, upon which the RDN technique was optimized, the feature selection procedure used in this case (i.e., top 20 features selected by ReliefF) was applied to the benchmark datasets. This potentially avoids background confounding that might perturb the effect of the AD method being applied to a given dataset.

For the calculation of the RDN AD for the two benchmark models, STD was used as provided in the OChem platform (calculated using the same method as described in this paper). As the output probabilities of each model of the ensemble were not available for the benchmark models, the agreement values were calculated from the inverse of the difference between average predicted probability and the observed value (so, an average predicted ensemble probability of 0.23 for an observed class value of 0 equates to 1 − |0 − 0.23| = 0.77 agreement). Even though this is more skewed than the frequency of correct predictions, it still represents the majority vote (or the overall predictive trend), to some extent. In fact this is a more conservative way to calculate the agreement since larger agreement values are only achieved when the majority of the predictions also have a value close to the expected class, and it is no longer sufficient that the majority is merely beyond (above or below) a threshold of P = 0.5.

Note that, to allow a closer analysis of the rate at which data is being included at each iteration of each AD method, all AD profiles will be presented as Accuracy as a function of amount of included data into the AD. As different AD techniques often generate different types of threshold values (number of neighbours, standard deviation, and density percentile), this standardization also allows a simpler and more intuitive visual analysis of the readouts. However attention must be paid to the fact that the actual establishment and use of each technique relies solely on the output measures. So, two profiles for the same technique applied to the same dataset under different parameters (e.g., a different set of features) might generate a percentage of 15 and 70% of included data, respectively, within their first iteration. If this first iteration is measuring the average distance to the first nearest neighbour, both cases will compute this distance differently (due to the use of different parameters), which will in turn generate a larger or smaller inclusion of data.

## Results and discussion

### The role of feature selection in establishing the RDN AD for P-gp dataset

**Fig. 6:**
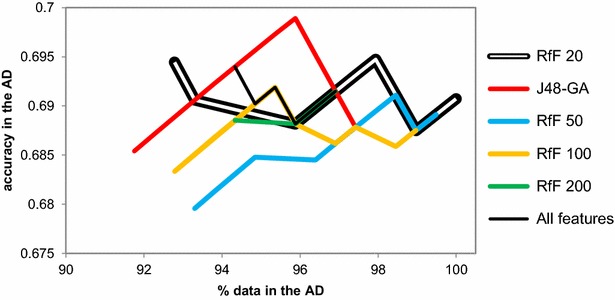
Comparison of different feature sets used in the dk-NN AD by Sahigara et al. [12], applied to the P-gp IV set

Firstly, the original dk-NN was implemented on the IV set using different sets of features to assess the impact of different sizes of the feature set. Figure 6 shows very different AD curves for different features used. Interestingly, the feature set leading to the best IV performance in P-gp model development [23], namely J48-GA-derived features, revealed to be far from acceptable for AD characterization using this technique, as the smallest distance around the AD core includes almost the entire dataset (91.8% coverage) and it shows an accuracy of 0.685, which is below the baseline accuracy of the global IV set at 0.691. This is in line with the theoretical expectation that the training of the QSAR model and the calculation of the AD are two different tasks, as already explained in the “Methods” section.

**Fig. 7:**
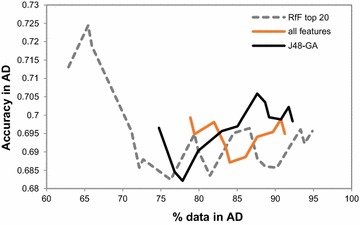
Comparison between RDN applied to the P-gp IV dataset using RfF top 20 features, all features or features selected by J48-GA. *Note* that this implementation of RDN corresponds to using the distances as directly from the k-average nearest neighbour (i.e., the distance shrinking to 1/3 and 1/2 has not been applied yet at this point, as explained later in the discussion)

The AD profiles built from all features and from RfF top 20 features were the best ones showing signs of decreasing degradation as the distance to the model’s core increases. As this indicates the possible ability of these two feature sets to locate higher quality predictions at the model’s core, both feature sets, namely the RfF top 20 features and all features, were tried in the RDN AD development as well as the model’s feature sets, J48-GA, for comparison (see Fig. 7). Figure 7 shows that by using RfF top 20 features a better resolution is achieved at the model’s core. More precisely, using all features leads to the inclusion of ~80% of the external data at the first iteration, while using RfF top 20 features, only ~62% of the data is included in the first iteration. Also, both the RfF top 20 and J48-GA curves show a statistically significant difference (Wilcoxon paired signed rank test, P = 0.0270, carried at a 95% confidence level after a failed Shapiro–Wilk normality test). Despite this, the improvement by ReliefF is around 1% compared with other methods and therefore, although statistically significant, this may be also due to the bias inherited from the dataset, therefore more validation is needed.

In addition, the RDN AD developed by using RfF top 20 features shows a visible decline in accuracy as the distance to model’s core is increased (by addition of new data). This shows an improvement when compared with dk-NN AD developed by this same set of molecular descriptors (compare Figs. 6 and 7). This means that penalising the distance thresholds attributed to each training instance according to their reliability (measured in STD and agreement) is useful towards mapping an AD with a higher quality core.

Results show that neither of the feature options commonly used in AD development—i.e., the model’s descriptors or all available descriptors [6, 32]—were appropriate for this dataset. The lack of ability to differentiate high reliability regions and low reliability regions across the chemical space when using all features is probably a sign of an overwhelming amount of noise that prevents the algorithm from taking advantage of meaningful variables. This goes against expert recommendation that all available features should be used [32]. Even if these observations do not necessarily apply to each and every QSAR problem, they should at least raise awareness to the fact that a feature selection routine should be carried within the task of AD characterization.

It would be theoretically expected that J48-GA would lead to a better AD characterization as it yielded a better learning performance which, in practice, means that it generated a decision tree better able to differentiate the two classes. However, the herein reported results show that ReliefF was visibly better able to generate more informative features with respect to misprediction–correct prediction separation (Fig. 7). Considering that classification errors happen by lack of ability to differentiate the two classes at certain regions of the chemical space, it is possible that features that directly address class differentiation are more explanatory in these problematic locations of the structure–activity landscape.

The reason why ReliefF outperforms J48-GA in this particular task might be because it selects relevant features even if they are highly correlated to other highly ranked features [20, 21]. This is possibly advantageous when defining the AD as two features might be highly correlated but still necessary to provide chemical coverage at specific locations of the data, which can be interpreted as feature cooperation—recall that feature combinations can potentially hold information that an isolated feature cannot show, as exemplified by Dragos et al. [6] (highly correlated hydrogen bond donor capacity and (positive) charge provide potentially essential information when combined). This ability to capture local idiosyncrasies and to uncover informative label interactions are some of the strongest characteristics of ReliefF [18, 20, 33], and it has been recommended as useful when the task can take advantage of strong feature interactions [20.]

In addition, using a wrapper means the bias of the J48-GA feature selection algorithm interacts with the bias of the J48 learning algorithm [34, 35]. Tetko et al. [13] reported that using the descriptors previously used to train the model does not lead to a better AD. This is in line with our observations that the features used for the modelling did not yield the best AD. Given that ReliefF generated high quality AD for the benchmark dataset (discussed below), we propose this technique is, in principle, particularly well-suited for AD mapping.

### Implementation of the RDN-AD using ReliefF top 20 feature set

**Fig. 8:**
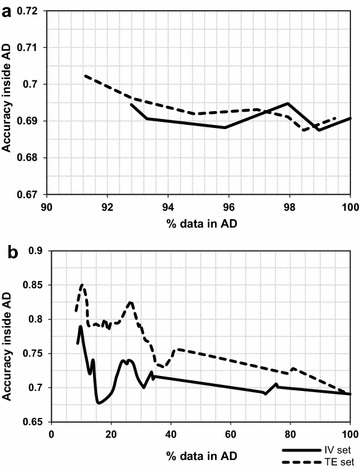
Comparison between dk-NN (**a**) and RDN (**b**) AD, both computed using the top 20 ReliefF selected features applied to the P-gp dataset. RDN was implemented with different distance increase steps as explained in the “Methods” section

Even though using ReliefF top 20 features yielded a visible improvement in the AD quality, Fig. 7 shows that, at this point, the RDN technique is still insufficient in mapping the reliability close to the model’s core, as taking into account the region up to the average 1st nearest neighbour satisfies more than 60% of IV data. Hence it can be deduced that the supposed inner-most region of the AD is far too large to be able to sort predictions for their reliability. This led to the implementation of three different distance steps as the neighbourhoods are increased (as described in the “Methods” section). We have hypothesized that, as regions closer to the AD’s core are expected to have more data, this area requires smaller steps for increasing distance, and as distances to the training data get larger the step can also increase. Applying this modification in distance step size did in fact bring a marked improvement in the quality of the AD core, as depicted in Fig. 8 by the higher accuracy value at the first iterations of ReliefF top 20. As explained before, recall that the percentage of included data is a mere result of an underlying distance-to-model threshold measure. As a result, the first point in both profiles corresponds to the same iteration (which in this case is the respective average distance to the first nearest neighbour). Additionally to this, ReliefF top 20 also yielded better resolution at the AD’s core (a smaller portion of data included at the first iteration, which allows a more gradual monitoring of quality across chemical space).

Furthermore, there is a marked difference between the initial dk-NN-derived profiles and the final RDN profiles (Fig. 8, A vs B). Considering that the dk-NN method can be regarded as the backbone of the RDN technique, this marked improvement in the ability to sort external set predictions according to their reliability is attributed to taking into account the local bias and precision (the correction factors), as well as allowing a slower increase of the AD span (i.e., slower scanning from the core to the outer regions of chemical space).

Figure 8b shows that even though the accuracy *vs* size of the AD is not a smooth profile, it shows a very similar trend between the two external sets (IV and TE sets). There is a main accuracy drop in the RDN AD at around 15% of data in the AD, which corresponds to a specific Euclidean distance from every training instance. So, it is probable that the chemical space corresponding to instances that fall around this distance is problematic. As a consequence, more importantly than having perfectly smooth profiles of degradation with respect to distance to the model, it is a priority that the established AD profile (in this case through the IV set) is able to correctly characterize how new data will behave, in a robust manner, across chemical space. One should remember that other issues of the model are being brought along with any AD assessment, i.e., activity cliffs, experimental errors in the response variable, and specific shortcomings of the machine learning task undertaken (e.g., overfitting).

Note that the percentage of inclusion and accuracy are cumulative. So, as the model space is being further explored, whenever an unreliable region is reached the detrimental effect of poor accuracy associated with compounds in this region will be propagated to the following regions, and their accuracy values will be deteriorated. This means that, when a low quality patch is found around the area corresponding to 15% of included data, this will decrease the accuracy at the following regions, which means that quality at the location of 23% inclusion would actually be higher than the observed 74%.

In an attempt to establish the cause for the abrupt decline observed at the beginning of the AD curve in Fig. 8b, we analysed the compounds entering the AD around 15% of included data. The descending part of the curve that precedes this point corresponds to 4 compounds being added through 4 distance steps (4 iterations of the algorithm), which in itself indicates this is a sparse region of the model. As a consequence, it is understandable that 3 of those 4 instances are mispredicted, given the theoretical link between data density and predictive confidence. It would be very difficult for the model to properly establish any link between structure and activity dependence with such scarcity of information on both aspects.

Looking into the absolute maximum (model’s core) of the AD, it was observed that the 18 molecules covered at this point are generally very dissimilar (similarity matrix in Additional file 1: Figure S1), showing a 0.1137 median Tanimoto coefficient of ECFP4 fingerprints, which spanned between 0.029 and 0.71. This rules out the assumption that the model’s core corresponds to a cluster of data—which would render this AD very limited for new data; instead the model’s core is spread across chemical space, into various smaller sub-portions of the core.

**Fig. 9:**
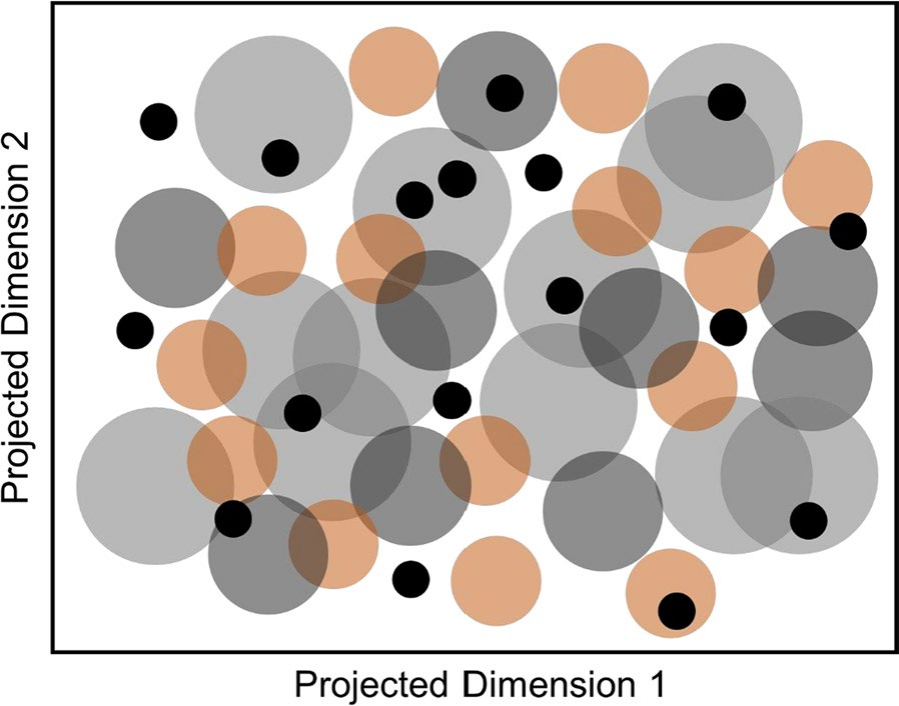
Visual representation of the RDN AD across two projected dimension of the input set of molecular descriptors. *Larger* (*light gray*) *circles* are established from training instances with higher density and/or higher reliability (small bias and large precision), and as *circles* decrease is size (*dark gray*, and *orange*) this indicates less dense/reliable regions of training space. External test predictions (*black*) are placed onto chemical space and if covered by any of the training circles they are deemed as being within the AD, for the established distance threshold

Figure 9 shows a graphical depiction of the neighbourhood circles around the training space, and how the external set scatters with respect to it.

### Comparison between RDN and STD AD

Ensemble standard deviation (STD) and STD-related methods are arguably some of the most successful AD techniques in the literature [27] (see comparative studies in [6, 7, 13]). As a result we have selected STD as our “gold standard” comparator, and comparisons will be made with respect to TE set performance, and degree of matching between IV set and TE set.

**Fig. 10:**
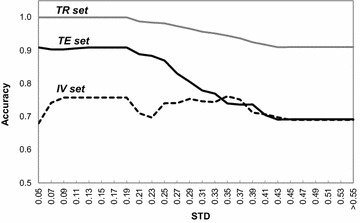
Accuracy across STD tiers for the different P-gp datasets

Figure 10 shows the STD AD profile for TR, IV and TE sets as a plot of accuracy versus the standard deviation between the ensemble predictions. Firstly it is important to note how misleading it is to use the training set to define the AD, as commonly done by QSAR practitioners. As clearly shown in Fig. 10, the training set gives an overly optimistic reliability profile across STD, which stems from the natural tendency for overfitting, and also possibly due to the systematic bias for the external sets. In this scenario, it is preferable to have a conservative reliability profile given by the IV set, which is what we have done with the RDN AD above.

**Fig. 11:**
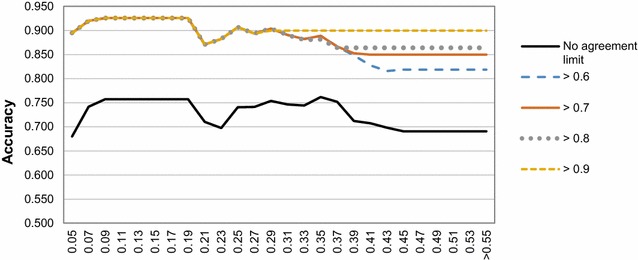
STD AD taking into account different agreement levels in the P-gp IV dataset

Even though STD shows a very smooth profile on the TE set, this does not mean that STD outperforms RDN, as the addition of new compounds is based on the standard deviations of predictions by various ensemble models, which is a more supervised procedure than RDN (Fig. 8b) where compounds were being added based on the corrected distance to training data. In addition, Fig. 10 shows that there is a marked difference between TE set and IV set accuracy profiles across AD, which renders this technique unpredictable with new data. This difference stems from the fact that low STD does not necessarily mean high quality of prediction, and it merely translates into high precision of the machine learning task—the lack of sensitivity to bias is the main flaw of this method, which is addressed in the newly proposed RDN method through the addition of the weighting term *W*_*i*_ (which accounts for both). Therefore, different datasets suffer, to different extents, from systemic bias when training a QSAR model. This phenomenon can be demonstrated by the notable impact that accounting for bias (by using the agreement measure) has in both profile smoothness and inner-core quality (Fig. 11). If agreement is taken into account, situations of high precision-high bias (affecting the quality of the STD AD) are overcome for the IV set. This observation further supports the use of both precision and bias measures as correction factors in the RDN algorithm.

The RDN performs similarly to the STD method in terms of the similarity between the accuracy profiles of both P-gp external datasets (IV and TE sets), the two profiles show a similar trend where degradations of performance occur around the same points in the X-axis (compare Figs. 8b and 10).

**Fig. 12:**
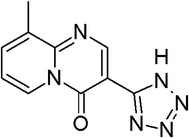
Example of an external set compound (Pemirolast) for which prediction is misleadingly reliable based on the STD method. However, the RDN correctly associated this with low-reliability prediction, which matches the misprediction outcome observed for this compound

To demonstrate the utility of RDN, let us consider one of the compounds with the lowest ensemble STD scores in our test set (Pemirolast, shown in Fig. 12, has an STD of 0.0284). According to its STD score, this compound would be deemed very reliably predicted, however it is actually systematically mispredicted. In contrast to STD, the RDN applicability domain only covers this compound at around 70% data coverage. As a result RDN is effectively able to overcome this systematic bias and correctly identify this as a lower-reliability prediction.

As RDN AD describes a consistent relationship between distance-to-model (or RDN distance) and accuracy in two external datasets, it should be used as a measure of prediction confidence across the chemical space, rather than merely a single point AD threshold where some compounds are included while others are excluded. Hence, instead of assigning compounds as in- or out-of-domain, they should be associated with different prediction confidences. This is a more sensible use for the AD, as it would be up to the end user to select the maximum acceptable error rate level. Furthermore, as shown by RDN and, to a lesser extent, by STD (Figs. 8, 10), this continuous AD characterization allows mapping the reliability landscape across the data. This can be used to identify problematic regions in the model, which is more productive than merely accepting or excluding predictions (as in the leverage AD, for example). For example, using Fig. 8b the predicted P-gp queries that fall in regions up to 13%, and between 22 and 27% of included data (which indicate an actual Euclidean distance) are expected to be more reliably predicted according to the AD profile. The AD profile also shows that from 70% inclusion onwards, there is a much higher probability of compounds being mispredicted.

Additionally, the impact of the minimum requirement for the number of training neighbours was investigated (ranging between 2 and 30 as described in the “Methods” section) and the results revealed no benefit from increasing the number of neighbours (see the Additional file 1, section “Impact of the minimum required number of training neighbours”).

### Complementary analysis with other AD: diagnosing mispredictions

**Fig. 13:**
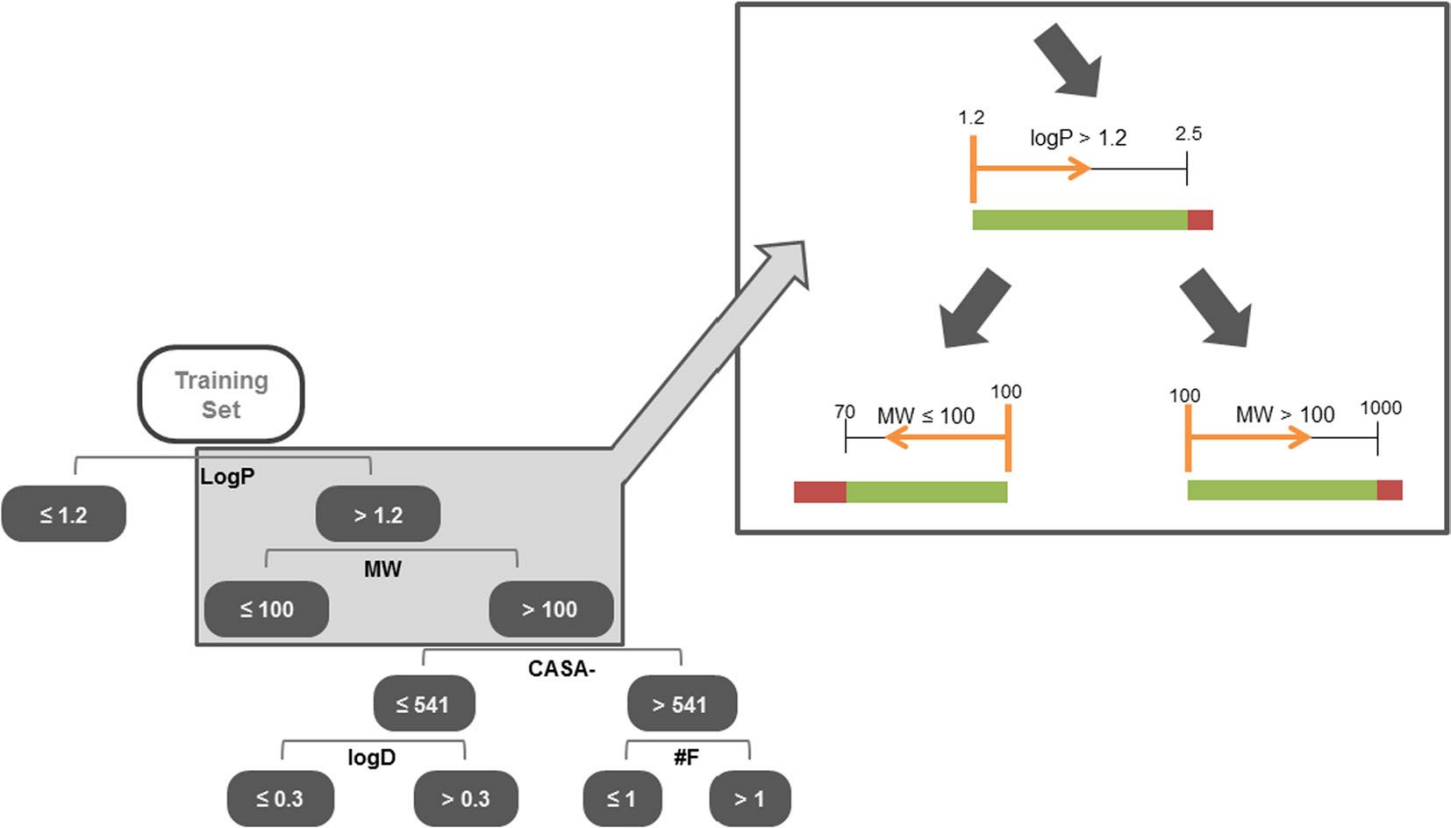
Schematics of the branch span assessment

Descriptor range has been used as a simple way of defining the applicability domain of a QSAR model. Here, in order to identify whether mispredictions are more commonly found outside the chemical span of the model, we computed the descriptor range of the training set compounds at each of the branches in the decision tree model. This strategy was previously proposed by Tong et al. [36], however, we limited descriptor range to the instances actually passing through each of the tree branches, instead of considering the descriptor range of the entire dataset. The rationale behind this experiment consists of the fact that a given tree ramification may, for example, establish that class 1 has MW > 100 g mol^−1^ and class 2 has MW ≤ 100 g mol^−1^, which are one-sided limits. This means that a query with MW = 50 g mol^−1^ is able to pass through that node even though the training cases that pass through the same node have MW ranging [70–100]. In reality, this compound is outside the range “known” by the trained model, and will be detected as such in this experiment (process illustrated in Fig. 13). Curiously, the 62 test instances that fell outside the respective branch’s descriptor range were associated with 72.6% accuracy, while the compounds inside the descriptor range showed 67.7% accuracy. This shows that falling outside training range is not necessarily the cause of misprediction. This justifies and further supports the use of an AD, like RDN, that identifies possible problematic regions within the data.

**Fig. 14:**
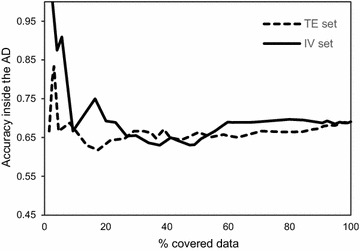
KDE results on IV and TE sets of the P-gp dataset

On the other hand, a method such as KDE, which is one of the most sophisticated AD approaches known for being able to detect empty regions in the data [8], also shows marked unpredictability in new data (Fig. 14). Its utility is based on the expectation that empty or less populated regions equate to weaker predictive performance due to insufficient chemical information. Figure 14 shows that the two external sets show different profiles (taking into account a comparison between the slopes of equivalent segments of both curves). This suggests that even looking at the inner space in descriptor range (which is the case with KDE method), as opposed to looking at the descriptor range, does not appear to be sufficient by itself, as density appears to relate to predictive accuracy in a non-robust manner (Fig. 14). However, the figure still shows some level of correlation between density and predictive performance. Low percentage of data coverage indicates higher density thresholds in the density plot across the first principal component (used to calculate the density distribution model), and as this threshold is decreased (the AD boundaries get expanded) there is an overall trend of decreasing accuracy. Nevertheless this is still a very rough trend, and the fact that accuracy does not evolve in the same manner in both datasets, as data coverage is increased, indicates that addressing data density is not sufficient as a standalone AD measure, but it could be a useful parameter towards characterization of a model’s AD. This corroborates the inclusion of this property in the RDN algorithm.

### Evaluation of RDN on benchmark datasets

To validate the utility of RDN, this was applied to two previous models built from benchmark data, Ames and CYP450. Note that the two benchmark datasets were modelled using neural network training, while we modelled the P-gp data with a decision tree method. Additionally, let us recall that the same feature selection method was used for all AD methods across all datasets (ReliefF top 20 features).

**Fig. 15:**
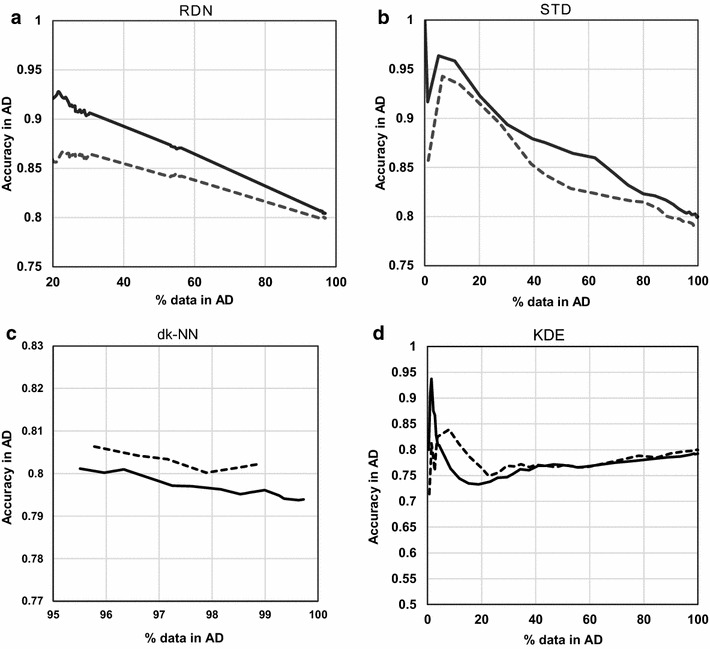
All four AD methods applied to the Ames model. Each of *both lines* in each graph corresponds to the same partition of the test set. Each *line* type represents one of the two external test sets from the Ames dataset; plot **a** shows the RDN method, **b** is the STD method, **c** is the dk-NN method and **d** is the KDE method

**Fig. 16:**
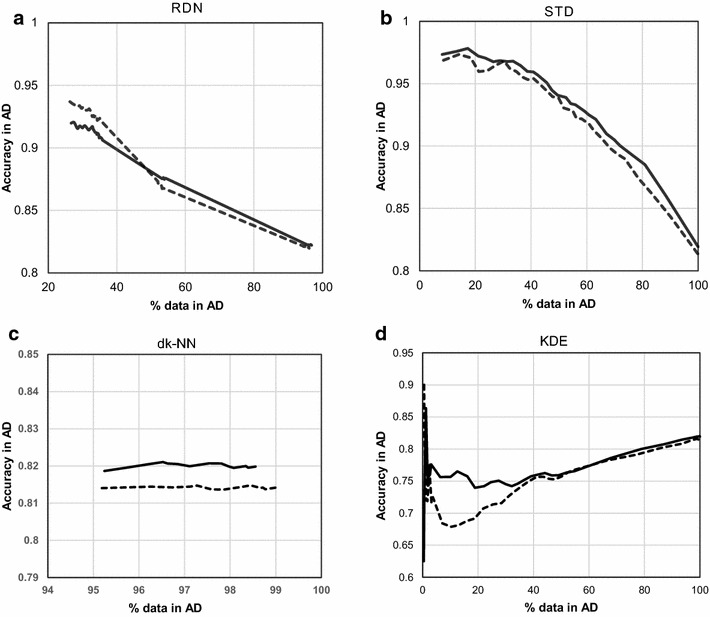
All four AD methods applied to the CYP450 model. Each of *both lines* in each graph corresponds to the same partition of the test set. Each *line* type represents one of the two external test sets from the CYP450 dataset; plot **a** shows the RDN method, **b** is the STD method, **c** is the dk-NN method and **d** is the KDE method

Both benchmark modelled datasets resulted in a smooth, decreasing curve of accuracy *vs* percentage of included data in the AD with RDN (which directly translates into distance to the model) (Figs. 15, 16). Furthermore, the shape of the curve in the two external datasets within each benchmark dataset is similar. In addition to RDN, Figs. 15 and 16 show that STD and dk-NN also generate curves of similar shape for the two external sets, however this was not the case for KDE. This reinforces the need to test a model’s AD in two different sets of data.

The main difference between RDN and STD with respect to the Ames model was that RDN profiles differed only in absolute accuracy values and maintained a similar overall curve shape for the two external sets, whereas STD revealed a significant difference in shape between the two curves at the core of the AD. This is very likely due to systematic bias in the model, which produces agreeing predictions in the ensemble which are consistently incorrect (i.e., a low STD for incorrect predictions). As in the RDN method both precision and bias are accounted for, this shortcoming has been overcome.

For CYP450 similar overall performance to that with Ames has been obtained. Moreover, in this case, both external subsets showed very similar absolute accuracy values. STD performed also very reliably with CYP450 but, once again, there is more oscillation of accuracy near the core of the model than with RDN. This oscillation is however not so marked that it would lead one to question STD’s robustness across other data. However, this is another example of a possible systematic bias that the ensemble STD could not overcome.

Results from both datasets confirm the validity of RDN as a method to appropriately define the applicability domain of a QSAR, by allowing a robust mapping of local predictive reliability across chemical space. Recall that this AD technique is completely independent from the model, and the AD is established solely using the training set. New predictions are merely sorted into different regions of the AD landscape after span of coverage around the training set has been set, at each iteration of the algorithm. The fact that correctly predicted instances show higher probability of being found near the training instances that are less biased and more precisely captured by the QSAR model demonstrates that, as theoretically expected, the reliability of a neighbourhood is inherited by its occupiers.

Furthermore, the independent role of density with respect to determining predictive reliability can be assessed by dk-NN and KDE as both sort the data according solely to density, where dk-NN does it at a local level, whereas KDE does it on a global scale. According to Figs. 15 and 16, both KDE and dk-NN methods fail to achieve a descending level of accuracy with distance from the model’s core. In addition, in both Ames and CYP450, the two different external subsets show different profiles, indicating that density and predictive performance vary unpredictably with respect to each other. As with the P-gp model, the Ames model also shows an overall slight descending trend with KDE and dk-NN. This supports the hypothesis that utilizing density information (both local and global) could play a role in the determination of a robust AD. On the other hand, the fact that the two CYP450 external datasets show quite different profiles with KDE, and this same technique has very different outcomes between all three datasets indicates that this method is not reliable as a standalone measure for AD determination and there may be other factors that should be taken into account. While global density appears to have an unpredictable role in predictive reliability, one cannot conclude that density has no role in the establishment of an AD, as when it is addressed at a local level in the dk-NN method it shows very low resolution at the core, which might be hiding meaningful correlations with accuracy.

## Assessment of the AD quality using a scoring function

Here we propose a scoring function to numerically measure the suitability/shortcomings of an AD curve (see the “Methods” section). Using this function leads to the same conclusions obtained from visual analysis of the AD profiles (scores are summarized in Table 1). According to the AD scoring function, Ames and CYP450 show more similar external set curves with RDN than with STD, which indicates RDN is in general a more robust way for AD profiling. On the other hand, KDE obtained the worst (highest) score in all three models. Despite what was previously established regarding the value of RDN, here the quality score points to the superiority of STD for the P-gp dataset. Recall that the quality score rewards the descending, smooth curves, and indeed STD has a smoother profile; however, RDN has the advantage of robustly locating poor quality regions (as discussed earlier). This shows that the scoring function may not necessarily follow the qualitative assessment of the AD profiles. Note that we do not mean to claim RDN is better performing than STD in all possible scenarios and datasets; instead, as with model development, the best AD method must be evaluated and the best method adopted in a case-by-case situation within every modelling effort. It is possible that some datasets suffer more from the effects of bias and hence they would benefit from RDN to overcome the systematic bias aspect of the STD method. This could explain why Ames and CYP450 models showed a very strong correlation between accuracy and distance to training space using RDN, and P-gp data shows a poorer trend.

**Table 1:** Summary of AD score across all three models studied

	AD score			
	RDN	STD	dk-NN	KDE
P-gp	4.40	*2.79*	6.82	8.14
Ames	*1.29*	1.92	4.48	9.26
CYP450	*1.01*	2.85	7.84	13.00

Lower AD scores (shown in italic) indicate a better scenario, translating into higher similarity to an ideal AD curve (smooth and decreasing trend of accuracy as a function of the AD span), and it also translates into a closely matching pair of two external set curves (which translates into the level of robustness)

As explained in the “Methods” section, in the calculation of the scoring function, the impact of any given sub-segment of the AD curves is corrected for the amount of data it is associated with. Consequently, even though visually all points in an AD curve carry the same weight, the proposed scoring scheme allows assigning the correct weight to each point according to the number of implicated instances. As a result, even though, in a comparison between CYP450-STD and CYP450-RDN, the AD characterization of the models with STD appears to be as robust as the RDN in the AD profile figures, STD it is in fact associated with more data being located in uncertain regions of chemical space.

In order to support the validity of this AD robustness score, it is worth analysing the contribution of simpler measures (or concepts) that are incorporated in the newly proposed score. Details of such analysis are available in the Additional file 1 (section “Complementary assessment of simpler curve similarity measures”), where it can be seen that none of the two parameters that constitute the proposed score, i.e., the pairwise similarity and the absolute difference between the curves, are sufficient on their own for assessing the quality of an AD profile, and the proposed scoring function is the most appropriate measure of AD robustness.

The fact that P-gp data is smaller and very noisy makes it more difficult for AD development. P-gp generated a poorer model (inferior test accuracy) [23], with a higher rate of mispredictions than Ames and CYP450 models, which makes the task of defining a smooth AD profile considerably harder. The noise in P-gp data comes from the variable threshold used in various sources to consider a compound as being a substrate [37] as well as the very large level of experimental uncertainty [38]. Furthermore, P-gp binding is notably known as being a very complex phenomenon driven by outstanding polyspecificity [39], which makes it naturally prone to error or bias in the experimental data.

## Conclusion

The utility of a QSAR relies on the theoretical assumption of a smooth relationship between independent features and the dependent variable [40], which allows its use for interpolations. However, as in reality the model’s landscape is not entirely smooth, it is crucial to map rugged regions across chemical space, since identifying these regions is the only way of assuring that the model is being safely used for future predictions [41.] The applicability domain establishes *where* the QSAR is smooth (i.e., where the dependency between structure and property holds). These rough “patches” in the structure–activity landscape could be due to input errors, abrupt changes in activity/property known as activity cliffs, or lack of chemical coverage due to data scarcity. We propose here that the adequate feature set optimized for the characterization of the AD can, in theory, reveal the problematic regions if the AD is optimized using external sets. By testing the AD performance with new data (external set), we increase the probability of having compounds falling in such “unseen” regions of structure–activity. As a result, the poor ability to predict these compounds will pinpoint the locations where the model should not be used. To address this issue, we introduced a novel AD characterization method that considers the impacts of local data density, as well as precision and robustness of predictions across the chemical space. In addition, we studied the role of feature selection paired with the AD technique, as opposed to the inheritance of feature selection previously carried for the model development.

The proposed new AD technique in this work, named RDN, is a hybrid technique, joining features from a density k-NN approach (which we called dk-NN) and the standard deviation of an ensemble model, as well as additional novel features like bias correction. The RDN AD allows taking into account: (1) sparse regions by mapping data density, as well as (2) local precision and bias. At the same time, we paired this method with ReliefF, which selects a set of molecular descriptors optimized to allow maximum separation between the classes to be predicted by the model. This method was applied to three different QSAR datasets and was compared with other established AD methods. Using the RDN AD allowed to improve the original distance-to-model method (dk-NN), which can be regarded as a simpler version of RDN. This improvement was visible through the increase of the accuracy at the core of the AD. RDN showed to be a robust AD technique that maintains an expected profile where performance degrades with increasing distance to the model in an external set. This technique showed overall better performance in comparison with the established STD method, as well as when compared with KDE, across all three datasets with a very strong correlation with accuracy.

Our results indicate that a given applicability domain needs to be assessed by the use of more than one external dataset to investigate the robustness of the AD. The two external sets can be compared in terms of accuracy *vs* distance-to-model profiles to indicate the reliability of a proposed AD. We also presented a scoring function to assess the quality of a given AD. The scoring function takes into account both robustness and the strength of the correlation with accuracy. As a result we propose the assessment of robustness as a standard procedure during the characterization of an AD, which can be done by evaluating the similarity of the relationship between accuracy and an AD measure for the two external subsets. This is a paramount aspect to take into account; without this there is no indication that a given AD can maintain its established accuracy profile across chemical space with new data.

In this work we challenge the common notion that either the QSAR model’s features or the entire feature set must be utilized for the establishment of the AD, and propose that a separate feature selection task should be performed specifically for AD development. Due to its particular characteristics, ReliefF has been proposed as a very effective algorithm for this. Results of this work showed that the feature set leading to the highest predictive performance is not necessarily the most adequate feature set for AD characterization. The proposed implementation of a feature selection routine using ReliefF showed to be successful in mapping accuracy across the structure–activity landscape.

Overall the RDN technique showed to effectively map prediction reliability across a QSAR model’s chemical space, and shows to be a useful tool to guide users on their decision regarding compound prioritization, thus promoting the user’s trust with the utility of the QSAR itself. This work helps reinforce the central role of AD characterization in any modelling workflow, as we demonstrate the importance of a thorough implementation and characterization of the AD.

## Additional file

**Additional file 1.** Results of supporting data analyses summarized in Figures S1, S2 and Tables S1–S3.

## Abbreviations

AD: applicability domain
dk-NN: density k-nearest neighbours
DTM: distance to model
IV: internal validation (set)
KDE: kernel density estimation
MW: molecular weight
P-gp: P-glycoprotein
QSAR: quantitative structure–activity relationships
RDN: reliability density neighbourhood
SMP: slope mismatch penalty
STD: standard deviation
TE: test (set)
TR: training (set)
