# Supplementary material for: A novel applicability domain technique for mapping predictive reliability across the chemical space of a QSAR: reliability-density neighbourhood
Source: J Cheminform. 2016 Dec 3;8:69. doi: 10.1186/s13321-016-0182-y (PMC5395519; doi:10.1186/s13321-016-0182-y)
Supplement: Supplementary file 1 — Additional file 1. Results of supporting data analyses summarized in Figures S1, S2 and Tables S1–S3. [file 13321_2016_182_MOESM1_ESM.docx]

**Supporting Information**

**Figure S1**. Model core analysis using a similarity matrix that is built with the Tanimoto coefficients of the ECFP4 fingerprints between each of the 18 compounds found at this region. Each of the compounds enumerated at the similarity matrix are depicted bellow.

| ID | 1 | 2 | 3 | 4 | 5 | 6 | 7 | 8 | 9 | 10 | 11 | 12 | 13 | 14 | 15 | 16 | 17 | 18 |
| --- | --- | --- | --- | --- | --- | --- | --- | --- | --- | --- | --- | --- | --- | --- | --- | --- | --- | --- |
| 1 |  |  |  |  |  |  |  |  |  |  |  |  |  |  |  |  |  |  |
| 2 |  |  |  |  |  |  |  |  |  |  |  |  |  |  |  |  |  |  |
| 3 |  |  |  |  |  |  |  |  |  |  |  |  |  |  |  |  |  |  |
| 4 |  |  |  |  |  |  |  |  |  |  |  |  |  |  |  |  |  |  |
| 5 |  |  |  |  |  |  |  |  |  |  |  |  |  |  |  |  |  |  |
| 6 |  |  |  |  |  |  |  |  |  |  |  |  |  |  |  |  |  |  |
| 7 |  |  |  |  |  |  |  |  |  |  |  |  |  |  |  |  |  |  |
| 8 |  |  |  |  |  |  |  |  |  |  |  |  |  |  |  |  |  |  |
| 9 |  |  |  |  |  |  |  |  |  |  |  |  |  |  |  |  |  |  |
| 10 |  |  |  |  |  |  |  |  |  |  |  |  |  |  |  |  |  |  |
| 11 |  |  |  |  |  |  |  |  |  |  |  |  |  |  |  |  |  |  |
| 12 |  |  |  |  |  |  |  |  |  |  |  |  |  |  |  |  |  |  |
| 13 |  |  |  |  |  |  |  |  |  |  |  |  |  |  |  |  |  |  |
| 14 |  |  |  |  |  |  |  |  |  |  |  |  |  |  |  |  |  |  |
| 15 |  |  |  |  |  |  |  |  |  |  |  |  |  |  |  |  |  |  |
| 16 |  |  |  |  |  |  |  |  |  |  |  |  |  |  |  |  |  |  |
| 17 |  |  |  |  |  |  |  |  |  |  |  |  |  |  |  |  |  |  |
| 18 |  |  |  |  |  |  |  |  |  |  |  |  |  |  |  |  |  |  |

|  |  |  |  |  |  |  |  |  |  |  |  |  |  |  |
| --- | --- | --- | --- | --- | --- | --- | --- | --- | --- | --- | --- | --- | --- | --- |
| 0 | 0.1 | 0.15 | 0.2 | 0.25 | 0.3 | 0.35 | 0.4 | 0.45 | 0.5 | 0.55 | 0.6 | 0.65 | 0.7 | 0.75 |

Table S1. Summary of the average Slope Mismatch Penalty (SMP) and the Area Between Curves (ABC) across all three models studied. The SMP was calculated as explained in the methods section, and the ABC was calculated from the sum of the area of trapezoids formed between the two curves under analysis. The best value in each dataset is highlighted in grey.

|  | Average SMP | | | | Area Between Curves (ABC) | | | |
| --- | --- | --- | --- | --- | --- | --- | --- | --- |
|  | RDN | STD | dk-NN | KDE | RDN | STD | dk-NN | KDE |
| P-gp | 6.8 | 3.6 | 4.6 | 7.2 | 1.9 | 1.9 | 0.05 | 1.7 |
| Ames | 6.7 | 3.7 | 5.5 | 8.1 | 1.9 | 0.4 | 0.1 | 0.9 |
| CYP450 | 7.1 | 3.0 | 7.3 | 9.2 | 0.5 | 0.4 | 0.1 | 1.1 |

***Impact of the minimum required number of training neighbours***

The results presented regarding RDN consider an external compound within the AD if it falls within the threshold distance of at least 1 training compound at any given iteration of the algorithm (refer to scheme 1, where at the last step new instances will be considered as being covered if falling within “any MaxDist*_i_*”, meaning within at least 1 training neighbourhood). To explore the impact of this parameter, the effect of increasing the minimum number of required nearest neighbours was tested. Except for a required minimum of 2 nearest neighbours (2 NN_min_), increasing the number of training neighbours revealed to be useless, yielding a low quality AD core often worse than the baseline accuracy achieved when all data are considered. Imposing a restriction of 2 NN_min_ showed higher quality at the inner most region of the model (Figure S2) compared to when one single neighbour is required (1 NN_min_) (Figure 8, Results and Discussion), but on the other hand the obtained profiles from the latter were smoother.

As a result, it is not straightforward to choose one alternative over the other. However, this experiment showed that, counterintuitively, having 1 neighbour as minimum requirement does not only provide a useful AD but it is better than, say, 4 nearest neighbours. This is in line with our remaining observations that point towards the importance of addressing small regions in the chemical landscape.

Figure S2. RDN AD with minimum 2 nearest neighbours required in order for a query to be considered included in the AD.

**Complementary assessment of simpler curve similarity measures**

To complement the analysis of the AD scoring function other simpler measures were analysed. A pairwise similarity was calculated based on comparing every sub-section between the curves for the two external subsets, and counting the percentage of matching segments (in terms of slope) between a pair of curves (Table S2). In all datasets this measure produces at least one occasion where higher pairwise similarity does not correspond to a visually better profile. This could be explained by the fact that purely looking at the similarity between curves does not distinguish between descending and increasing trends, which led us to conclude that absolute similarity in itself is insufficient in assessing the quality of an AD profile. Another evaluated measure, which is a part of the AD scoring function, is the SMP. This could be considered as a more sophisticated pairwise similarity, as it takes into account both slope mismatch and slope direction (results summarized in Table S3). In this case, the lower the value, the better is the overall trend between both external curves. The average SMP is consistently lower with STD across all datasets, however as already explained this could be misleading as matching slopes between a pair of curves can have a different value according to the amount of data associated with each section.

Lastly, the Area Between Curves (ABC) (Table S3) shows that all three datasets have the smallest ABC with dk-NN, which again shows this would be misleading to use as an assessment measure. Solely having a small absolute difference between both curves does not necessarily mean the AD profile has more quality, as shown by Ames dk-NN AD where the two curves are close to each other, yet they have a very poor characterization of the model’s AD.

**Table S2**. Pairwise similarity across all three models studied. Pairwise Similarity indicates the percentage of segments in both external set curves which show a matching slope. The best value in each dataset is highlighted in grey.

|  | pairwise Similarity (%) | | | |
| --- | --- | --- | --- | --- |
|  | RDN | STD | dk-NN | KDE |
| P-gp | 50 | 50 | 68 | 55 |
| Ames | 45 | 71 | 46 | 64 |
| CYP450 | 55 | 88 | 46 | 63 |

Table S3. Summary of the average Slope Mismatch Penalty (SMP) and the Area Between Curves (ABC) across all three models studied. The SMP was calculated as explained in the methods section, and the ABC was calculated from the sum of the area of trapezoids formed between the two curves under analysis. The best value in each dataset is highlighted in grey.

|  | Average SMP | | | | Area Between Curves (ABC) | | | |
| --- | --- | --- | --- | --- | --- | --- | --- | --- |
|  | RDN | STD | dk-NN | KDE | RDN | STD | dk-NN | KDE |
| P-gp | 6.8 | 3.6 | 4.6 | 7.2 | 1.9 | 1.9 | 0.05 | 1.7 |
| Ames | 6.7 | 3.7 | 5.5 | 8.1 | 1.9 | 0.4 | 0.1 | 0.9 |
| CYP450 | 7.1 | 3.0 | 7.3 | 9.2 | 0.5 | 0.4 | 0.1 | 1.1 |
